# Sanguinarine improved nutrient digestibility, hepatic health indices and productive performance in laying hens fed low crude protein diets

**DOI:** 10.1002/vms3.436

**Published:** 2021-02-11

**Authors:** Masoumeh Bavarsadi, Amir Hossein Mahdavi, Saeed Ansari‐Mahyari, Elaheh Jahanian

**Affiliations:** ^1^ Department of Animal Sciences College of Agriculture Isfahan University of Technology Isfahan Iran

**Keywords:** crude protein, egg cholesterol, laying hens, liver health indices, performance, Sanguinarine

## Abstract

A major mean to minimize feeding costs and faecal nitrogen excretion on poultry farms is to decrease the supplied dietary protein content. This, however, is associated with the declines in productive performance and systemic health indices. Sanguinarine may improve protein efficiency via decreasing the intestinal amino acid decarboxylation and stimulating the tryptophan‐serotonin pathway. The present study was carried out to investigate the effects of dietary supplementation of sanguinarine on the performance, egg yolk biochemical parameters, serum enzyme activities, nutrient digestibility, ovarian follicles, and hepatic health indices in laying hens fed decremental levels of crude protein (CP). For this purpose, 180 laying hens were allocated into nine dietary treatments with four replicates of five birds each. The experimental treatments consisted of three levels of CP (85.0%, 92.5%, and 100% of Hy‐Line W‐36 manual recommendation) and three levels of sanguinarine (0.00, 3.75, and 7.50 mg/kg) in a 3 × 3 factorial arrangement administered during a 70‐day feeding trial. Results showed that the decremental levels of CP led to significant increases in serum aspartate aminotransferase (*p* < .05), alanine aminotransferase, and alkaline phosphatase (*p* < .01) activities, egg yolk cholesterol concentration (*p* = .064), and hepatic fat and malondialdehyde (MDA) contents (*p* < .05). It also caused the significant declines in ileal dry matter (DM) digestibility (*p* < .05) and eggshell strength (*p* < .05), and also tended to decrease CP digestibility (*p* = .071), Haugh unit (*p* = .057) and egg production percentage (*p* = .062). The interaction effects of the experimental factors indicated that dietary supplementation of sanguinarine, especially at 7.50 mg/kg, led to significant improvements in serum aspartate aminotransferase and alanine aminotransferase activities (*p* < .01), egg yolk cholesterol (*p* < .001) and triglyceride (*p* < .05) concentrations, eggshell strength (*p* < .001), Haugh unit (*p* < .05), hepatic fat (*p* < .001) and MDA (*p* = .059) contents, ileal DM and CP digestibility (*p* < .01) as well as egg production, egg mass and feed conversion ratio (FCR; *p* < .05) in birds receiving decremental levels of CP. Taken together, the results indicate that dietary administration of sanguinarine could enhance productive performance via improving nutrient digestibility, hepatic health indices and fortifying systemic antioxidant capacity in laying hens fed low‐CP diets.

## Graphical abstract

Decremental levels of CP increased hepatic enzymes activity, fat percentage and MDA content. Decreasing CP content lowered DM and CP digestibility, and egg production percentage. Sanguinarine suppressed egg yolk cholesterol and triglyceride in laying hens fed on low‐CP diet. Sanguinarine improved performance, antioxidant capacity and hepatic health indices.

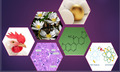

## 1 INTRODUCTION

A direct relationship has been found between dietary crude protein (CP) level and N excretion. Moreover there is a growing global concern over environmental pollution due to the excessive urinary and faecal excretion of protein and nitrogen (Praes et al., 2014). Hence, it is essential to practice novel strategies to decrease nitrogen excretion and dietary CP content; in turn, they minimize feeding costs on poultry farms (Abbasi et al., 2014). A decrease in dietary CP content, however, caused some disadvantages which are related to the reductions in productive performance and also systemic health indices (Malomo et al., 2013). A novel remedy to overcome this problem is the use of herbal products that might reduce dietary CP requirement via improving protein efficiency.

One such aromatic plant that has recently attracted a lot of attention for its strong antioxidant activity is *Macleaya cordata*. The most important substance in sangrovit^®^ extracted from the rhizome of this herb is a benxophenanthridine alkaloid compound (Blank et al., 2010) called “sanguinarine” that possesses antibacterial (Bavarsadi et al., 2017), antifungal (Mellor, 2001), antioxidant (Lee et al., 2015), anti‐inflammatory (Tanaka et al., 2001) and immunomodulatory (Mellor, 2001) properties. Furthermore, it is known to inhibit the intestinal aromatic amino acid decarboxylase (Dršata et al., 1996; Mellor, 2001; Vieira et al., 2008), and to improve the synthesis of serotonin in the liver, intestine and brain tissue (Mellor, 2001). This inhibitory action of sanguinarine is due to the similarity of its molecular structure to those of aromatic amino acids such as tryptophan, phenylalanine and tyrosine inducing sanguinarine molecules to bind to the L‐amino acid decarboxylase and, thereby becoming irreversibly blocked (Dršata et al., 1996). In this way, sanguinarine increases the availability of aromatic amino acids (Vieira et al., 2008) leading to the reductions in toxic biogenic amines concentrations (Dršata et al., 1996). Thus, the improvement in dietary protein efficiency as the consequence of dietary sanguinarine supplement might be ascribed to the lower amounts of unabsorbed peptides and amino acids passing into the lower gastrointestinal tract (Juśkiewicz et al., 2005).

Notably, the administration of sanguinarine in diet is far from clear; because controversial results have been reported on the effects of its dietary inclusion on animal performance. Vieira et al. (2008) observed that administration of 37.5 mg/kg sangrovit improved feed intake in broilers given the decremental CP percentages. Similarly, Blank et al. (2010) noted that dietary supplementation of sangrovit at 50 mg/kg improved growth performance in pigs fed diets low in tryptophan. However, Matulka et al. (2014) reported that dietary inclusion of *Macleaya cordata* extract had no significant effects in terms of live weight, body weight gain and feed intake in chicks. Additionally, dietary sangrovit supplementation at 30 mg/kg was observed to have no impact on caecal ammonia concentration in broiler chicks (Juśkiewicz et al., 2013).

Given the scant information and contradictory results regarding the effects of dietary inclusion of sanguinarine on egg productivity and nutrient digestibility in laying hens, the present study was undertaken to assess the effects of different levels of this bioactive substance on the performance, egg quality, egg yolk biochemical parameters, serum enzyme activities, nutrient digestibility, differential ovarian follicle counts and hepatic health indices in laying hens fed the decremental levels of CP.

## 2 MATERIALS AND METHODS

### 2.1 Experimental design and dietary treatments

For the purposes of this study, 180 white Leghorn laying hens, 40 weeks of age, were randomly assigned into nine experimental diets with four replicates of five birds each. Prior to the start of the experiment, a 30‐day pretest recording period was performed and finally laying hens were selected based on the same body weight and egg production percentage, as the test unit. Dietary treatments consisted of a 3 × 3 factorial arrangement of treatments with three levels of CP (85.0%, 92.5% and 100% of Hy‐Line W‐36 manual recommendation) and three levels of sanguinarine (0.00, 3.75 and 7.50 mg/kg, Sangrovit^®^, Phytobiotics, Futterzusatzstoffe GmbH) administered during a 70‐day feeding trial. The total experimental period lasted 80 days, including 10 days of adaptation and 70 days of main recording period. Water and experimental diets were supplied ad libitum.

### 2.2 Chemical analysis and measurements

Prior to the trial, the corn and soybean meal used in the experimental feeds were analysed for its dry matter (DM) (930.15), CP (984.13), ether extract (954.02), calcium (927.02) and phosphorous (935.59) contents according to AOAC (2006). The experimental treatments were formulated using the obtained values to meet all the nutritional requirements according to Hy‐Line W‐36 guidelines (Table 1).

**TABLE 1:** Composition and nutrient content of basal diets (43–52 weeks of age)

Item	100% Protein	92.5% Protein	85% Protein
Ingredients (g/kg)			
Corn	592	628	663
Soybean meal	244	212	181
Soybean oil	30.0	24.5	19.1
Monocalcium phosphate	15.8	16.2	16.3
Calcium carbonate	106	106	106
Common salt	2.40	2.40	2.30
Filler^a^	0.50	0.50	0.50
DL‐Methionine	1.80	0.10	0.40
L‐Lysine HCL	0.00	1.60	1.40
Na bicarbonate	2.50	3.70	5.00
Vitamin premix^b^	2.50	2.50	2.50
Mineral premix^c^	2.50	2.50	2.50
Calculated nutritional levels			
Metabolizable Energy (kcal/kg)	2,800	2,800	2,800
Lysine (g/kg)^d^	7.90	7.25	6.68
Methionine (g/kg)^d^	4.21	3.96	3.67
Methionine + Cysteine (g/kg)^d^	6.70	6.27	5.78
Threonine (g/kg)^d^	5.91	5.58	5.09
Available phosphorous (g/kg)	4.61	4.61	4.61
Analysed nutritional levels			
Crude protein (g/kg)	152	141	130
Crude fat (g/kg)	52.2	50.1	48.7
Crude fibre (g/kg)	29.1	28.7	28.5
Calcium (g/kg)	43.5	43.5	43.5
Moisture (g/kg)	101	105	104

^a^ Filler represented inert space (sand) in the diet to which sanguinarine was added at the expense of it.

^b^ Vitamin premix provided the following per kilogram of diet: vitamin A (from vitamin A acetate), 9,800 IU; cholecalciferol, 2,100 IU; vitamin E (from dl‐α‐tocopheryl acetate), 22 IU; riboflavin, 4.4 mg; nicotinamide, 40 mg; calcium pantothenate, 35 mg; menadione, 1.50 mg; folic acid, 0.80 mg; thiamine, 3 mg; pyridoxine, 10 mg; biotin, 1 mg; choline chloride, 560 mg; and ethoxyquin, 125 mg.

^c^ Mineral premix provided the following per kilogram of diet: Mn, 65 mg; Zn, 55 mg; Fe, 50 mg; Cu, 8 mg; I [from Ca (IO3)2•H2O], 1.8 mg; and Se, 0.30 mg.

^d^ All diets formulated on total amino acids.

### 2.3 Serum enzyme activities

At the end of the experiment, two hens were randomly selected from each replicate, bled via the wing vein, and the serum samples were collected and stored at −20°C until analysis. Serum enzymatic activities including aspartate aminotransferase (AST), alanine aminotransferase (ALT) and alkaline phosphatase (ALP) were measured according to the turbidimetric method (Pars Azmoon, Tehran, Iran).

### 2.4 Determination of egg yolk biochemical parameters

Using the method described in Mousavi et al. (2017), the yolk lipid was extracted from three eggs per replicate at the end of the trial to determine their triglyceride and cholesterol concentrations using a spectrophotometric quantitation kit at 520 nm and the colorimetric Libermann‐Burchard method at 560 nm, respectively.

### 2.5 Measurements of hepatic biochemical and histological changes

On day 70 of the trial, two hens were randomly selected from each replicate and humanly sacrificed to investigate the effects of treatments on their liver relative weights (expressed as percent of live body weight) and the likely hepatic biochemical and histological changes. For this purpose, tissue samples were collected and ground to determine their moisture, lipid, and protein contents (AOAC, 2006).

The thiobarbituric acid reactive substances (TBARS) method as explained by Mousavi et al. (2017) was used to assess hepatic malondialdehyde (MDA) levels. Briefly, liver samples were weighed (2 g), added to 50‐ml test tubes containing 18 ml of 3.86% perchloric acid and homogenized. The resultant homogenate was filtered through Whatman No. 1 filter paper, before the filtrate (2 ml) was blended with 2 ml of 20 mM TBA in distilled water and incubated in a boiling water bath for 30 min. The obtained mixture was reacted with MDA and MDA‐like substances to produce a pink pigment with an absorption maximum at 532 nm.

A segment, approximately 1 × 1 × 0.5 cm thick, was obtained from the right liver lobe of each bird to be used for light microscopic observations. The histological samples were fixed in 10% formaldehyde solution. Transverse sections, 5 µm in thickness, were then prepared using a microtome, to be subsequently stained with haematoxylin–eosin for examination under a light microscope, as described in Mousavi et al. (2017).

### 2.6 Differential counts of ovarian follicles

At the end of the experimental period, two birds were randomly selected and humanly sacrificed to remove their ovarian follicles evaluated by measuring their diameters, which classified them into large white follicles (LWF, 2–5 mm), small yellow follicles (SYF, 5–10 mm) and large yellow follicles (LYF, >10 mm) according to the classification, as described in Rahman Alizadeh et al. (2016).

### 2.7 Ileal nutrient digestibility

Ileal nutrient digestibility was determined according to the acid insoluble ash (AIA) marker method of McCarthy et al. (1974). Briefly, diets containing 5 g/kg of Celite were given as a source of AIA for 4 consecutive days from day 67 to day 70 of the trial. Then, two birds per replicate were humanly sacrificed at the end of the study period. Their ileal contents were directly collected into 80 ml sampling cups, frozen at −20°C, and subsequently analysed for their AIA content, according to the method described in McCarthy et al. (1974), while their DM and CP levels were determined according to the standard procedures of Association of Official Analytical Chemists (2006).

### 2.8 Performance and egg quality

Eggs were collected daily at 9:00 a.m., counted and weighed to determine egg mass throughout the trial period. Performance parameters such as egg weight, egg production percentage, egg mass, feed intake and feed conversion ratio (FCR) were determined at 35 days intervals on days 35 and 70 of the experiment. Additionally, three eggs per replicate were collected to determine the egg quality parameters including eggshell weight, eggshell strength, eggshell thickness, Haugh unit, yolk index and yolk colour.

### 2.9 Statistical analysis

All the data were analysed using ANOVA by the GLM procedures of SAS software (SAS Institute, 2001). The following model was postulated in the analysis of all the traits: *Y_ijk_ = μ + A_i_ + B_j_ + AB_ij_ + e_ijk_*, where *Y_ijk_* = observed value for a particular trait, *μ* = overall mean, *A_i_* = effect of the *i* ^th^ level of sanguinarine, *B_j_* = effect of the *j* ^th^ level of CP percentage, *AB_ij_* = the respective interaction of the *i* ^th^ and *j* ^th^ levels of dietary sanguinarine and CP percentage, and *e_ijk_* = random error associated with the *ijk* ^th^ recording. A probability value of *p* < .05 was described to be statistically significant. Additionally, *p*‐values between 0.05 and 0.10 was shown and described as a trend. In cases where the differences were significant, the means were compared using the Tukey test. In addition, after normality had been examined with the Shapiro‐Wilk test, hepatic histological changes were analysed according to the GLM procedure of SAS software.

## 3 RESULTS

### 3.1 Egg quality

The decrease in dietary CP levels resulted in the decreased (*p* < .05) eggshell strength on days 35 and 70 of the trial. It tended to decrease eggshell thickness on day 70 (*p* = .068), and Haugh unit (*p* = .086; *p* = .057), as well as yolk colour (*p* = .071; *p* < .05) on days 35 and 70 of the experiment (Table S1). However, shell weight, shape index, and yolk index remained unaffected by the decremental levels of dietary CP.

Dietary supplemental sanguinarine only tended to increase eggshell strength on day 35 (*p* = .096) and 70 (*p* = .053) of the trial (Table S1).

Dietary CP and sanguinarine interactions revealed that using the highest sanguinarine level raised eggshell thickness (*p* = .051; *p* < .05), eggshell strength (*p* < .001; *p* < .001), and Haugh unit (*p* < .05; *p* < .05) on days 35 and 70 of the trial in the laying hens fed a low‐CP diet. In this group, supplementing 7.5 mg/kg of sanguinarine not only tended to enhance yolk colour on days 35 (*p* = .053) and 70 (*p* = .061) of the experiment but also it tended to increase eggshell weight on day 70 (*p* = .061) of the trial (CP × sanguinarine; Table 2).

**TABLE 2:** Effect of different levels of sanguinarine on egg quality traits in laying hens fed different levels of crude protein (CP)

	CP (%)									Probability			SEM^a^
	100			92.5			85			CP	Sanguinarine	CP* Sanguinarine	
Sanguinarine (mg/kg)	0.00	3.75	7.50	0.00	3.75	7.50	0.00	3.75	7.50				
Shell weight (g)													
35 days	8.58	8.83	9.02	8.38	8.61	8.88	8.42	8.73	8.92	0.565	0.247	0.204	0.294
70 days	8.71	8.98	9.30	8.61	8.83	9.00	8.48	8.71	8.89	0.473	0.409	0.061	0.293
Shell thickness (μm)													
35 days	37.6	38.1	38.4	37.3	37.6	37.9	37.1	37.3	37.6	0.136	0.258	0.051	0.366
70 days	37.2^a‐d^	37.7^a^	37.5^a^	36.6^a‐d^	37.0^a‐d^	37.2^a‐d^	36.4^a‐d^	36.7^a‐d^	37.0^a‐d^	0.068	0.200	0.017	0.293
Shell strength (kg/cm^2^)													
35 days	3.32^a‐d^	3.60^a^	3.72^a^	2.99^a‐d^	3.27^a‐d^	3.52^a‐d^	2.62^a‐d^	2.98^a‐d^	3.22^a‐d^	0.044	0.096	0.001	0.197
70 days	3.13^a‐d^	3.33^a‐d^	3.59^a^	2.85^a^	3.07^a‐d^	3.31^a‐d^	2.43^a‐d^	2.62^a‐d^	2.89^a‐d^	0.012	0.053	0.001	0.154
Shape index													
35 days	0.78	0.77	0.75	0.77	0.76	0.76	0.75	0.77	0.79	0.778	0.790	0.363	0.029
70 days	0.78	0.76	0.78	0.76	0.77	0.76	0.76	0.76	0.75	0.708	0.700	0.401	0.022
Haugh unit													
35 days	87.2^a‐d^	87.9^a^	88.7^a^	85.4^a^	86.1^a‐d^	86.9^a‐d^	84.4^a‐d^	85.1^a‐d^	86.0^a‐d^	0.086	0.201	0.038	1.174
70 days	86.9^a‐d^	87.6^a^	88.3^a^	85.8^a‐d^	86.7^a‐d^	87.3^a^	84.0^a‐d^	85.1^a‐d^	85.9^a‐d^	0.057	0.241	0.035	1.004
Yolk index													
35 days	0.43	0.44	0.45	0.42	0.43	0.44	0.40	0.42	0.43	0.625	0.694	0.237	0.032
70 days	0.44	0.44	0.45	0.43	0.44	0.44	0.42	0.43	0.43	0.603	0.735	0.226	0.021
Yolk colour													
35 days	7.00	7.50	7.00	6.50	6.25	6.50	6.25	6.50	7.00	0.071	0.518	0.053	0.254
70 days	7.50	7.00	7.25	7.50	7.00	7.25	6.25	6.00	6.00	0.046	0.547	0.061	0.153
Yolk cholesterol (mg/g egg yolk)	13.3^a‐d^	12.2^a‐d^	11.8^a‐d^	14.0^a‐d^	13.0^a‐d^	12.1^a‐d^	15.0^a^	13.7^a^	12.9^a‐d^	0.064	0.025	0.001	0.541
Yolk triglyceride (mg/g egg yolk)	232^a‐d^	228^a‐d^	221^a‐d^	237^a^	229^a^	2253^a‐d^	240^a^	236^a^	231^a^	0.239	0.193	0.018	4.172

^a^ SEM: standard error of the mean (*n* = 12 eggs).

^a‐d^ Means with no common superscript within each row are significantly (*p* < .05) different.

### 3.2 Egg yolk biochemical parameters

The decremental levels of CP tended to increase (*p* = .061) egg yolk cholesterol concentration. However, dietary sanguinarine supplementation, especially at the high dosage of 7.5 mg/kg, led to a significant (*p* < .05) decrease in egg yolk cholesterol content in hens (Table S1). Indeed, the supplementation of high sanguinarine level (7.5 mg/kg) significantly decreased not only egg yolk cholesterol (*p* < .001) but also triglyceride (*p* < .05) concentrations in the laying hens fed the low‐CP diet (Table 2).

### 3.3 Serum enzyme activities

The decline in dietary CP levels led to significant enhancements in serum AST (*p* < .05), ALT (*p* < .01) and ALP (*p* < .001) activities in the hens. In contrast, dietary supplementation of sanguinarine resulted in the marked reductions in serum ALT (*p* < .01) and ALP (*p* < .001) activities (Table S2). Similarly, dietary administration of sanguinarine, especially at its highest level (7.5 mg/kg), caused the improvements in serum AST (*p* < .01), ALT (*p* < .001) and ALP (*p* = .051) activities in birds supplied with the decremental CP levels (Table 3). However, dietary inclusion of sanguinarine at least 3.75 mg/kg level improved their serum AST, ALT and ALP activities in hens receiving the recommended CP level of Hy Line W36.

**TABLE 3:** Effect of different levels of sanguinarine on serum enzymes activities in laying hens fed different levels of crude protein (CP)

	CP (%)									Probability			SEM^a^
	100			92.5			85			CP	Sanguinarine	CP* Sanguinarine	
Sanguinarine (mg/kg)	0.00	3.75	7.50	0.00	3.75	7.50	0.00	3.75	7.50				
AST^b^ (U/L)	152^b^	150^a‐d^	147^a‐d^	168^b^	159^a‐d^	152^a‐d^	178^a^	170^a^	163^a^	0.048	0.151	0.005	6.075
ALT^b^ (U/L)	30.2^b^	25.4^a‐d^	19.3^a‐d^	29.2^b^	25.5^a‐d^	23.1^a‐d^	37.0^a^	34.3^a^	28.5^b^	0.005	0.002	0.001	1.566
ALP^b^ (U/L)	1,195	989	897	1,378	1,250	1,049	1,383	1,280	1,147	0.001	0.001	0.051	21.07

^a^ SEM: standard error of the mean (*n* = 8 birds).

^b^ AST: aspartate aminotransferase; ALT: alanine aminotransferase; ALP: alkaline phosphatase.

^a‐d^ Means with no common superscript within each row are significantly (*p* < .05) different.

### 3.4 Hepatic histological and biochemical alterations

The decreases in CP levels tended to decrease both hepatic colour density (*p* = .087) and tissue integrity (*p* = .057) but it resulted in significant enhancements in hepatic relative weight, fat percentage and MDA concentration (*p* < .05; Table S3). Dietary inclusion of the highest sanguinarine level (7.5 mg/kg) led to not only a marked increase in hepatic Kupffer cells numbers (*p* < .001) but also a considerable decrease in MDA concentration (*p* < .05), (Table S3). sangiuinarine supplementation especially at 7.5 mg/kg in laying hens supplied with decreasing dietary CP levels (CP × sanguinarine; Table 4), led to noticeable declines in hepatic relative weight (*p* < .05), tissue integrity (*p* < .01), fat percentage (*p* < .001) and also tended to reduce MDA content (*p* = .059).

**TABLE 4:** Effect of different levels of sanguinarine on liver relative weight (g/kg live body weight), and hepatic histological and biochemical changes in laying hens fed different level of crude protein (CP)

	CP (%)									Probability			SEM^b^
	100			92.5			85			CP	Sanguinarine	CP* Sanguinarine	
Sanguinarine (mg/kg)	0.00	3.75	7.50	0.00	3.75	7.50	0.00	3.75	7.50				
Liver relative weight	2.38^b^	2.34^b^	2.26^c^	2.56^c^	2.41^b^	2.33^c^	2.73^a^	2.59^a^	2.45^b^	0.048	0.089	0.011	0.061
Kupffer cell numbers (Score)^a^	3^+^	5^+^	5^+^	4^+^	4^+^	5^+^	4^+^	5^+^	5^+^	0.725	0.004	0.133	0.288
Colour density (Score)	4^+^	5^+^	5^+^	4^+^	5^+^	4^+^	4^+^	4^+^	4^+^	0.086	0.228	0.222	0.331
Tissue integrity (Score)	3^a^	5^a^	5^a^	5^a^	4^a^	4^a^	4^a^	4^a^	4^a^	0.057	0.405	0.004	0.288
Hepatic fat (mg/g)	225^c^	208^c^	185^a‐d^	252^a^	240^b^	222^c^	262^a^	250^a^	235^b^	0.041	0.199	0.001	1.055
MDA^c^ (mmol/L)	0.32	0.29	0.22	0.36	0.31	0.26	0.43	0.36	0.32	0.033	0.048	0.059	0.042

^a^ Number of +’s indicates severity of the histological changes.

^b^ SEM: standard error of the mean (*n* = 8 birds).

^c^ MDA: malondialdehyde.

^a‐d^ Means with no common superscript within each row are significantly (*p* < .05) different.

### 3.5 Ovarian follicle enumerations

The low‐CP diet showed no significant effects on the relative ovary weight or ovarian follicle (LWF, SYF and LYF) numbers in the experimental laying hens. Although dietary inclusion of the highest sanguinarine level led to a significant decrease (*p* < .05) only in ovarian LWF (2–5 mm) numbers (Table S4), these traits were remained unaffected by dietary sanguinarine supplementation in the laying hens receiving decreasing levels of CP (Table 5).

**TABLE 5:** Effect of different levels of sanguinarine on the relative weight of ovary (g/kg live body weight) and ovarian differential follicle numbers in laying hens fed different levels of crude protein (CP)

	CP (%)									Probability			SEM^b^
	100			92.5			85			CP	Sanguinarine	CP* Sanguinarine	
Sanguinarine (mg/kg)	0.00	3.75	7.50	0.00	3.75	7.50	0.00	3.75	7.50				
Ovary relative weight	0.49	0.56	0.61	0.49	0.50	0.56	0.48	0.49	0.58	0.707	0.381	0.686	0.063
LWF^a^ (2–5 mm)	40.0	35.7	31.0	35.2	33.7	29.5	33.7	31.7	28.2	0.211	0.049	0.379	2.090
SYF^a^ (5–10 mm)	20.2	18.7	16.6	18.2	16.5	15.2	17.7	15.2	14.7	0.474	0.398	0.115	2.281
LYF^a^ (>10 mm)	6.25	5.75	5.25	6.00	5.25	5.25	5.50	5.25	5.00	0.531	0.309	0.303	0.520

^a^ LYF: large yellow follicles; SYF: small yellow follicles; LWF: large white follicles.

^b^ SEM: standard error of the mean (*n* = 8 birds).

### 3.6 Ileal nutrient digestibility

The reductions in ileal DM (*p* < .05) and CP (*p* = .071) digestibility measurements were obtained in the hens fed the low‐CP diet (Table S5). Furthermore, ileal DM and CP digestibility were enhanced (*p* < .05) as a result of dietary inclusion of sanguinarine, especially at 7.5 mg/kg (Table S5). The interaction effects of the experimental factors indicated that additional dietary sanguinarine, particularly its highest level, led to remarkable improvements in ileal DM (*p* < .001) and protein (*p* < .01) digestibility in those hens receiving decreasing dietary CP levels (Table 6). Similarly, dietary supplementation of at least 3.75 mg/kg of sanguinarine significantly increased ileal DM and protein digestibility in hens receiving the recommended Hy‐Line W‐36 CP level (*p* < .01; Table 6).

**TABLE 6:** Effect of different levels of sanguinarine on illeal nutrient digestibility in laying hens fed different levels of crude protein (CP)

	CP (%)									Probability			SEM^a^
	100			92.5			85			CP	Sanguinarine	CP* Sanguinarine	
Sanguinarine (mg/kg)	0.00	3.75	7.50	0.00	3.75	7.50	0.00	3.75	7.50				
DM^b^ digestibility (%)	64.1^b^	66.5^a^	66.8^a^	63.0^a^	64.6^b^	65.1^b^	61.2^a‐c^	62.9^a‐c^	63.1^a‐c^	0.011	0.031	0.001	0.724
CP digestibility (%)	65.7^b^	68.7^a^	69.3^a^	64.5^a^	66.4^b^	67.9^b^	63.4^a‐c^	64.8^a‐c^	66.1^b^	0.071	0.047	0.003	0.971

^a^ SEM: standard error of the mean (*n* = 8 birds).

^b^ DM: dry matter.

^a‐c^ Means with no common superscript within each row are significantly (*p* < .05) different.

### 3.7 Performance

Declining CP levels led to significant decreases not only in egg production percentages both on day 35 (*p* < .05) and throughout the whole trial period (*p* = .062), but also in egg mass on day 35 (*p* = .059) and 70 (*p* = .092) of the experiment (Table S6). However, egg weight, feed intake and FCR remained unaffected in hens on the low‐CP diet. Dietary administration of the highest sanguinarine level resulted in the elevated egg production percentage on day 35 (*p* < .05) and tended to increase egg production percentage (*p* = .097) on day 70. This is, while, sanguinarine exhibited no effects on egg weight, egg mass, feed intake and FCR throughout the trial (Table S6). Dietary supplementation of the highest level of sanguinarine (7.5 mg/kg) led to significant rises in egg production percentages on days 35 (*p* < .01), 70 (*p* = .054), and throughout the whole trial period (*p* < .05), egg weight (*p* = .05) on day 35 of the trial, egg mass on day 35 (*p* < .001), 70 (*p* < .05), and the whole trial period (*p* < .05), feed intake (*p* < .05) on day 35, and the improvement in FCR on day 35 (*p* < .05) and the whole trial period (*p* < .05) in those laying hens receiving the decremental CP levels (Table 7). Interestingly, dietary inclusion of at least 3.75 mg/kg was associated with the positive effects on performance in those hens receiving the recommended CP levels of Hy‐Line W‐36.

**TABLE 7:** Effect of different levels of sanguinarine on performance in laying hens fed different levels of crude protein (CP)

	CP (%)									Probability			SEM^a^
	100			92.5			85			CP	Sanguinarine	CP* Sanguinarine	
Sanguinarine (mg/kg)	0.00	3.75	7.50	0.00	3.75	7.50	0.00	3.75	7.50				
Egg production (%)													
1–35 days	83.6^b^	85.9^b^	86.8^a^	82.4^a^	83.1^b^	84.7^b^	79.8^a‐c^	80.3^a‐c^	82.0^a‐c^	0.039	0.046	0.009	1.132
36–70 days	81.7	83.9	84.1	79.3	80.9	81.7	79.0	79.9	80.5	0.104	0.482	0.054	1.775
1–70 days	82.7^b^	84.9^a^	85.5^a^	80.9^a^	82.0^b^	83.2^b^	79.4^a‐c^	80.1^a‐c^	81.2^b^	0.062	0.097	0.036	1.313
Egg weight (g)													
1–35 days	62.9	62.8	64.3	61.0	62.7	63.1	60.2	62.1	62.8	0.392	0.321	0.050	1.315
36–70 days	64.2	65.2	65.5	63.8	63.3	64.6	62.5	63.3	63.9	0.613	0.864	0.302	1.903
1–70 days	63.5	64.0	64.9	62.4	63.0	63.9	61.4	62.7	63.4	0.572	0.651	0.142	1.758
Egg mass (g/day per bird)													
1–35 days	52.6^b^	54.0^a^	55.8^a^	50.3^a^	52.1^b^	53.5^b^	48.0^a‐c^	49.9^a‐c^	51.5^b^	0.059	0.103	0.001	1.531
36–70 days	52.5^b^	54.7^a^	55.1^a^	50.6^b^	51.2^b^	52.8^b^	49.4^a‐c^	50.6^b^	51.4^b^	0.092	0.425	0.013	1.551
1–70 days	52.5^a^	54.4^a^	55.5^a^	50.5^b^	51.7^b^	53.1^b^	48.7^a‐c^	50.2^a‐c^	51.5^b^	0.109	0.394	0.015	1.835
Feed intake (g/day per bird)													
1–35 days	95.5^b^	96.9^a^	97.7^a^	94.0^a^	95.6^a^	95.0^b^	93.4^a‐c^	94.4^b^	95.2^b^	0.169	0.471	0.012	1.153
36–70 days	95.7	93.4	94.1	91.9	91.9	93.8	90.3	91.0	91.6	0.691	0.625	0.201	1.910
1–70 days	95.6	95.1	95.9	92.9	93.8	94.4	91.9	92.7	93.4	0.375	0.599	0.118	1.625
FCR^b^ (g of feed: g of egg)													
1–35 days	1.82^b^	1.79^b^	1.75^a‐c^	1.87^a‐c^	1.83^b^	1.78^b^	1.94^a^	1.89^a^	1.85^a^	0.194	0.218	0.040	0.062
36–70 days	1.82	1.71	1.71	1.82	1.79	1.78	1.83	1.80	1.78	0.406	0.728	0.270	0.081
1–70 days	1.82^a^	1.75^b^	1.73^b^	1.85^a^	1.81^a^	1.78^b^	1.89^a^	1.85^a^	1.82^a^	0.325	0.409	0.048	0.050

^a^ SEM: standard error of the mean (*n* = 20 birds).

^b^ FCR: feed conversion ratio.

^a‐c^ Means with no common superscript within each row are significantly (*p* < .05) different.

## 4 DISCUSSION

### 4.1 Egg quality

The low‐CP diet in this experiment decreased not only eggshell thickness and strength but also Haugh unit and yolk color index. It, however, had no effects on eggshell weight, egg shape, and yolk indices (Table 2). Consistent with our findings, Novak et al. (2008) reported the depression in albumen percentage and the enhancement in yolk colour in their laying hens supplemented with the low‐CP diet. Similarly, Adeyemo et al. (2012) showed that decreasing CP content (to 140 g/kg) led to remarkable decreases in albumin index, yolk index, eggshell thickness and Haugh unit. Nevertheless, some reports found no effects of decreasing dietary CP content on these traits (Praes et al., 2014). These findings indicate that a minimum CP content is necessary to prepare essential amino acids not only for optimum eggshell mineralization but also for matrix biosynthesis (Adeyemo et al., 2012).

Although dietary sanguinarine supplement was found to increase eggshell strength, it showed no effects on other egg quality traits. The improved eggshell strength was observed as a consequence of sanguinarine supplementation might be ascribed to its inhibitory effect on the oxidative burst enzyme (i.e., NADPH oxidase) in the uterus leading to the reduction in free radicals levels (Slaninová et al., 2014; Vrba et al., 2004). It is considered that herbal bioactive components stimulate the enzymatic pathways of protein synthesis in the uterine eggshell glands, thus improving eggshell strength by concentrating collagen in the shell matrix (Mirbod et al., 2017). In addition, sanguinarine reportedly released calcium ions from the sarcoplasmic reticulum, resulting in the improved eggshell mineralization (Hu et al., 2001).

### 4.2 Egg yolk biochemical parameters

Declining CP levels tended to increase egg yolk cholesterol concentration in the experimental laying hens. This is possibly related to the undesirable effects of reducing dietary CP content on hepatic health indices and fatty liver syndrome induction that give rise to cholesterol synthesis in the liver (Table 4). In this regard, Swennen et al. (2007) noticed that low‐CP diets stimulated hepatic lipogenesis, leading to the enhancement in triglycerides and lipoproteins excretion into the blood stream (Hada et al., 2013).

Additional dietary sanguinarine levels were found to decrease egg yolk cholesterol concentration. The inhibitory effects of the bioactive compounds exited in herbal medicines on hepatic 3‐hydroxy‐3‐methyl‐glutaryl‐CoA reductase (i.e., the rate‐limiting enzyme of cholesterol biosynthesis) might be responsible for the observed lower egg yolk cholesterol content (Srinivasan, 2005). Our findings are consistent with those of previously reports that indicated dietary inclusion of medicinal plants or their extracts decreased egg yolk cholesterol content (Mirbod et al., 2017; Mousavi et al., 2017). The interaction effects of the experimental factors indicated that both yolk cholesterol and triglyceride concentrations were decreased as a consequence of supplementing low‐CP diets with sanguinarine. The inhibition of Acetyl‐CoA synthesizing enzyme and, thereby, the fatty acid synthesis complex by botanic bioactive substances might be accountable for the observed reduction in egg yolk triglyceride content (Qureshi et al., 1983).

### 4.3 Serum enzyme activities

A decrease in dietary CP levels led to increases in serum AST, ALT and ALP activities in laying hens. This might be associated with an increase in hepatic fat percentage (Table 4). Because these enzymes, known as hepatic health indices (Akbarian et al., 2012), have been shown to increase as the consequence of liver injuries or high fat accumulation in the liver. Consistent with our results, Dairo et al. (2010) observed that serum AST and ALT activities increased in broilers fed low energy and CP diets.

Supplemental sanguinarine was observed to reduce serum ALT and ALP activities in laying hens. Sanguinarine reportedly inhibits a broad range of enzymes, especially those possessing active sulfhydryl groups including Na‐K ATPase, ALT and AST (Dršata et al., 1996). Sanguinarine in its imine form plays an important role in either oxidant scavenging or enzyme inhibition (Dostál & Slavík, 2002). Our findings are also in line with those of Jankowski et al. (2009), who showed that dietary supplementation of the *Macleaya cordata* alkaloid extract resulted in the decreased serum AST activity and thiobarbituric acid reactive substances in broiler chicks. Similarly, Matulka et al. (2014) showed that the feeding 500 mg/kg of *Macleaya cordata* extract led to a significant decrease in serum alkaline phosphatase activity, but it did not affect serum AST and ALT activities in chicks.

Dietary inclusion of the highest sanguinarine level was found to be capable of improving serum AST, ALT and ALP activities in hens receiving decremental CP levels, whereas administration of at least 3.75 mg/kg sanguinarine was observed to decrease the activities of these serum enzymes in hens receiving the recommended CP level. Hepatoprotective effect of sanguinarine seems to be triggered into action not only with a normal CP diet supplemented with the minimum sanguinarine level but also with the decremental CP diet supplemented with its maximum level.

### 4.4 Hepatic biochemical and histological alterations

Feeding the low‐CP diet tended to decrease hepatic colour density and tissue integrity, but it elevated the hepatic relative weight, fat percentage and MDA concentration. The higher hepatic relative weight and fat percentage might have been due to the higher serum triglyceride content (Bavarsadi et al., 2017) leading to the higher fat accumulation in the liver. These findings are confirmed by Bunchasak et al. (2005), who observed an increase in hepatic triglyceride concentration in hens on diet with 18% CP level. It has been demonstrated that nutrition participates in the development of several hepatic syndromes, since the liver plays a key role in lipid metabolism owing to its involvement in lipid degradation, storage or lipogenesis (Hada et al., 2013; Swennen et al., 2007). The observed enhancement in hepatic MDA content relative to the control group might be associated with the higher lipid accumulation in the hepatocytes of hens fed low‐CP diets, suggesting that overproduction of free radicals in low‐CP diets plays a role in tissue integrity repression.

Dietary administration of sanguinarine at least 3.75 mg/kg level caused the more hepatic Kupffer cells in the hens. Niu et al. (2012) showed that sanguinarine not only inhibited the expression of inflammatory mediators but also decreased expressions of intracellular adhesion molecule‐1 and vascular cell adhesion molecule‐1. It may, thus, be claimed that sanguinarine was able to induce peripheral immunological reactions (Tanaka et al., 2001). Similarly, the results of our previous study showed that the percentage of monocytes in the blood exhibited a numerical increase in laying hens receiving dietary sanguinarine supplement (Bavarsadi et al., 2017).

The interaction effects of the trial factors showed that the dietary sanguinarine supplement, especially at 7.5 mg/kg level, led to considerable decreases in hepatic relative weight, tissue integrity, fat percentage and MDA content in laying hens fed on the decreasing dietary CP content. Jankowski et al. (2009) reported that the presence of dietary supplementation of the *Macleaya cordata* alkaloid extract caused a reduction in the relative liver weight in chicks. In agreement with our observations, Lee et al. (2015) reported that dietary supplementation of 50 mg/kg sangrovit resulted in the reduced MDA concentration in thigh meat of broilers. This might be attributed to the antioxidant activity of sanguinarine, as also previously noted by Vrba et al. (2004), Slaninová et al. (2014), and Bavarsadi et al. (2017).

### 4.5 Ovarian follicle enumerations

The decremental levels of dietary CP affected neither the relative ovary weight nor the ovarian follicle counts in the experimental hens. This is while dietary inclusion of the highest sanguinarine level led to a decline in the ovarian LWF numbers. Angiogenesis is a vital event in the growth of ovarian follicles (Fraser & Wulff, 2001). Administration of high levels of sanguinarine is reported to suppress not only the production of vascular endothelial growth factor but also the vascular endothelial growth factor‐induced Akt activation in granulose cells, thereby probably leading to the decrease in vessel growth in the ovarian follicle (De Stefano et al., 2009). However, this bioactive substance has remarkable modulatory effects on both local and systemic inflammatory reactions. Sanguinarine plays its anti‐inflammatory role not only through inhibiting lipoxygenase and cyclooxygenase‐1 activities but also via mobilizing arachidonic acid‐induced Ca^2+^ (Jeng et al., 2007).

### 4.6 Ileal nutrient digestibility

Decreasing dietary CP levels were found to lower the ileal DM and CP digestibility in the experimental birds (Table S5). More specifically, dietary CP content was observed to affect nitrogen intake and excretion, which are related to dietary nutrient availability (Praes et al., 2014). The ileal DM digestibility was highly reliant on dietary protein levels; so that the lower dietary CP level resulted in the lower ileal DM digestibility (Dahlman et al., 2002). In agreement with our results, Novak et al. (2008) showed that decreasing dietary CP content reduced protein digestibility in their laying hens.

Dietary sanguinarine supplementation was found to improve the ileal digestibility of both DM and CP in the experimental hens (Table 6). A number of explanations might be claimed for the observed increase in nutrient digestibility upon sanguinarine intake. For instance, the rise in the amounts of nutrients was made available due to the reduction in ileal microbial counts (Bavarsadi et al., 2017). Another mechanism to explain the enhancement in nutrient digestibility is an increase in the secretion of digestive enzymes of the gastrointestinal tracts as a result of dietary inclusion of sangrovit (Franz et al., 2005). Finally, sanguinarine was shown to improve intestinal function in absorbing water and nutrients including ingested protein and amino acids (Liu et al., 2013).

### 4.7 Performance

Dietary inclusion of the decremental CP levels declined egg production percentage and egg mass, whereas it had no effects on egg weight, feed intake or FCR in the birds. Our findings are in agreement with previous reports (Bunchasak et al., 2005; Novak et al., 2008; Praes et al., 2014) suggesting the detrimental effects of feeding low‐CP diets on egg production might be ascribed to the induced lower values of ileal DM and CP digestibility (Table 6) as well as lower intestinal villi height to crypt depth ratio (Bavarsadi et al., 2017). Similar to our observations, Rashid et al. (2004) observed that diets with the decremental CP levels up to 150 g/kg led to the decreases in egg production, egg weight, egg mass and feed efficiency in crossbred hens as compared with those fed on high‐protein diets. Adeyemo et al. (2012) reported that although a reduction in CP content (140 g/kg) resulted in the decreases in egg yield percentage and egg weight and an elevation in FCR, it had no influence on feed intake in their hen layers. Unlike ours, some studies have shown that feed intake and egg weight were decreased after feeding low‐CP diets (Bunchasak & Silpasorn, 2005).

The interaction effects of the experimental factors indicated that dietary inclusion of sanguinarine improved feed intake, egg mass and FCR in those laying hens receiving the decremental dietary CP levels (Table 7). In agreement with our findings, Yakhkeshi et al. (2011) reported that dietary inclusion of sangrovit improved FCR in broiler chicks in both the finisher and total experimental periods. Teillet et al. (2012) indicated that although dietary inclusion of 80 mg/kg sangrovit lowered FCR in rabbits, it had no effects on their growth rate. Pickler et al. (2013) noted that dietary inclusion of sanguinarine at 50 mg/kg level led to the improvement in FCR in chicks. Our findings are in line with those reported by Lee et al. (2015), who observed that administration of 50 mg/kg sangrovit improved feed intake during the grower phase in broilers. The observed improvements in the productive performance of hens fed on the low‐CP diets (85.0%, 92.5% of Hy‐Line recommended CP levels) supplemented with the highest level of sanguinarine might be explained by recourse to the following two factors: (1) the improvement in dietary protein efficiency achieved through reducing the intestinal decarboxylation of aromatic amino acids induced by the inhibition of L‐aminoacid decarboxylase (Dršata et al., 1996), and (2) the enhancement in feed consumption as a result of stimulating the tryptophan‐serotonin pathway (Mellor, 2001). Furthermore, these responses might have been promoted by the suppression of gut harmful microflora and, simultaneously, by the improvements achieved in the intestinal health indices (Bavarsadi et al., 2017; Juśkiewicz et al., 2013). These might have collectively increased nutrient utilization and absorption in the laying hens on diets supplemented with sanguinarine.

## 5 CONCLUSION

The present study concluded that dietary administration of sanguinarine is capable of enhancing productive performance via improving nutrient digestibility, hepatic health indices and fortifying systemic antioxidant capacity in laying hens fed low‐CP diets.

## CONFLICT OF INTEREST

All authors declare no conflict of interests.

## ETHICAL STATEMENT

All the experimental proceedings in this experiment were approved by the Animal Care and Use Committee of Isfahan University of Technology (IUT), Isfahan, Iran.

## ANIMAL WELFARE STATEMENT

The authors confirm that the ethical policies of the journal, as noted on the journal's author guidelines page, have been adhered to and approval via the Animal Care and Use Committee of Isfahan University of Technology and Federation of Animal Science Societies under protocol (FASS, 2010) has been received. The authors confirm that they have followed EU standards for the protection of animals used for scientific purposes.

### PEER REVIEW

The peer review history for this article is available at https://publons.com/publon/10.1002/vms3.436.

## Supporting information

Supplementary Material
