# Supplementary material for: Sanguinarine improved nutrient digestibility, hepatic health indices and productive performance in laying hens fed low crude protein diets
Source: Vet Med Sci. 2021 Feb 11;7(3):800–11. doi: 10.1002/vms3.436 (PMC8136927; doi:10.1002/vms3.436)
Supplement: Supplementary file 1 — Supplementary Material [file VMS3-7-800-s001.docx]

**Supporting Information**

**Sanguinarine improved nutrient digestibility, hepatic health indices and productive performance in laying hens fed low crude protein diets**

**Running title:** Sanguinarine and dietary CP on laying hens performance

**Table S1** *The main effect analysis of* *different levels of sanguinarin on egg quality traits in laying hens fed different levels of crude protein (CP)*

|  |  | | | | | | | | | | Probability | | |
| --- | --- | --- | --- | --- | --- | --- | --- | --- | --- | --- | --- | --- | --- |
|  | CP (%) | | |  | Sanguinarine (mg/kg) | | |  |  | CP | | Sanguinarine | SEM^a^ |
|  | 100 | 92.5 | 85 |  | 0.00 | 3.75 | 7.50 |  |  |  | |  |  |
| Shell weight (g) |  |  |  |  |  |  |  |  |  |  | |  |  |
| 35 d | 8.81 | 8.62 | 8.70 |  | 8.46 | 8.72 | 8.94 |  |  | 0.565 | | 0.247 | 0.294 |
| 70 d | 9.00 | 8.81 | 8.69 |  | 8.60 | 8.84 | 9.06 |  |  | 0.473 | | 0.409 | 0.293 |
| Shell thickness (μm) |  |  |  |  |  |  |  |  |  |  | |  |  |
| 35 d | 38.0 | 37.6 | 37.3 |  | 37.3 | 37.6 | 37.9 |  |  | 0.136 | | 0.258 | 0.366 |
| 70 d | 37.5 | 36.9 | 36.7 |  | 36.7 | 37.1 | 37.3 |  |  | 0.068 | | 0.200 | 0.293 |
| Shell strength(kg/cm^2^) |  |  |  |  |  |  |  |  |  |  | |  |  |
| 35 d | 3.55^a^ | 3.26^ab^ | 2.94^b^ |  | 2.98 | 3.28 | 3.49 |  |  | 0.044 | | 0.096 | 0.197 |
| 70 d | 3.35^a^ | 3.08^b^ | 2.65^c^ |  | 2.8 | 3.01 | 3.26 |  |  | 0.012 | | 0.053 | 0.154 |
| Shape index |  |  |  |  |  |  |  |  |  |  | |  |  |
| 35 d | 0.77 | 0.76 | 0.77 |  | 0.77 | 0.77 | 0.76 |  |  | 0.778 | | 0.790 | 0.029 |
| 70 d | 0.77 | 0.76 | 0.76 |  | 0.77 | 0.76 | 0.76 |  |  | 0.708 | | 0.700 | 0.022 |
| Haugh unit |  |  |  |  |  |  |  |  |  |  | |  |  |
| 35 d | 88.0 | 86.1 | 85.2 |  | 85.7 | 86.4 | 87.2 |  |  | 0.086 | | 0.201 | 1.174 |
| 70 d | 87.6 | 86.6 | 85.0 |  | 85.5 | 86.5 | 87.3 |  |  | 0.057 | | 0.241 | 1.004 |
| Yolk index |  |  |  |  |  |  |  |  |  |  | |  |  |
| 35 d | 0.44 | 0.43 | 0.42 |  | 0.42 | 0.43 | 0.44 |  |  | 0.625 | | 0.694 | 0.032 |
| 70 d | 0.44 | 0.43 | 0.43 |  | 0.43 | 0.44 | 0.44 |  |  | 0.603 | | 0.735 | 0.021 |
| Yolk color |  |  |  |  |  |  |  |  |  |  | |  |  |
| 35 d | 7.17 | 6.42 | 6.58 |  | 6.58 | 6.75 | 6.83 |  |  | 0.071 | | 0.518 | 0.254 |
| 70 d | 7.25^a^ | 7.25^a^ | 6.08^b^ |  | 7.08 | 6.67 | 6.83 |  |  | 0.046 | | 0.547 | 0.153 |
|  |  |  |  |  |  |  |  |  |  |  | |  |  |
| Yolk cholesterol (mg/g egg yolk) | 12.4 | 13.1 | 13.9 |  | 14.1^a^ | 13.0^b^ | 12.3^b^ |  |  | 0.064 | | 0.025 | 0.541 |
| Yolk triglyceride (mg/g egg yolk) | 227 | 230 | 236 |  | 236 | 231 | 226 |  |  | 0.239 | | 0.193 | 4.172 |

^a^SEM: standard error of the mean (n= 12 eggs).

^a-c^ Means with no common superscript within each row are significantly (P<0.05) different.

**Table S2** *The main effect analysis of* *different levels of sanguinarin on serum enzymes activities in laying hens fed different levels of crude protein (CP)*

|  |  | | | | | | | | | Probability | | |
| --- | --- | --- | --- | --- | --- | --- | --- | --- | --- | --- | --- | --- |
|  | CP (%) | | |  | Sanguinarine (mg/kg) | | |  |  | CP | Sanguinarine | SEM^a^ |
|  | 100 | 92.5 | 85 |  | 0.00 | 3.75 | 7.50 |  |  |  |  |  |
| AST^b^ (U/L) | 150^b^ | 161^b^ | 172^a^ |  | 166 | 159 | 154 |  |  | 0.048 | 0.151 | 6.075 |
| ALT^b^ (U/L) | 25.0^b^ | 25.9^b^ | 33.2^a^ |  | 32.1^a^ | 28.4^b^ | 23.7^c^ |  |  | 0.005 | 0.002 | 1.566 |
| ALP^b^ (U/L) | 1027^c^ | 1226^b^ | 1271^a^ |  | 1319^a^ | 1174^b^ | 1032^c^ |  |  | 0.001 | 0.001 | 21.07 |

^a^SEM: standard error of the mean (n= 8 birds).

^b^AST: aspartate aminotransferase; ALT: **alanine aminotransferase; ALP:** alkaline phosphatase.

^a-c^ Means with no common superscript within each row are significantly (P<0.05) different.

**Table S3** *The main effect analysis of* *different levels of sanguinarin on liver relative weight (g/kg live body weight), and hepatic histological and biochemical changes in laying hens fed different level of crude protein (CP).*

|  |  | | | | | | | | | | Probability | | |
| --- | --- | --- | --- | --- | --- | --- | --- | --- | --- | --- | --- | --- | --- |
|  | CP (%) | | |  | Sanguinarine (mg/kg) | | |  |  | CP | | Sanguinarine | SEM^b^ |
|  | 100 | 92.5 | 85 |  | 0.00 | 3.75 | 7.50 |  |  |  | |  |  |
| Liver relative weight | 2.33^b^ | 2.44^b^ | 2.59^a^ |  | 2.56 | 2.45 | 2.35 |  |  | 0.048 | | 0.089 | 0.061 |
| Kupffer cell numbers (Score)^a^ | 4^+a^ | 4^+^ | 4^+^ |  | 3^+b^ | 5^+a^ | 5^+a^ |  |  | 0.725 | | 0.004 | 0.288 |
| Color density (Score) | 5^+^ | 4^+^ | 4^+^ |  | 5^+^ | 4^+^ | 4^+^ |  |  | 0.086 | | 0.228 | 0.331 |
| Tissue integrity (Score) | 5^+^ | 4^+^ | 4^+^ |  | 4^+^ | 4^+^ | 4^+^ |  |  | 0.057 | | 0.405 | 0.288 |
| Hepatic fat (mg/g) | 206^b^ | 238^ab^ | 249^a^ |  | 246 | 233 | 214 |  |  | 0.041 | | 0.199 | 1.055 |
| MDA^c^ (mmol/L) | 0.27^b^ | 0.31^ab^ | 0.37^a^ |  | 0.37^a^ | 0.32^ab^ | 0.27^b^ |  |  | 0.033 | | 0.048 | 0.042 |

^a^Number of +’s indicates severity of the histological changes.

^b^SEM: standard error of the mean (n= 8 birds).

^c^MDA: malondialdehyde.

^a-b^ Means with no common superscript within each row are significantly (P<0.05) different.

**Table S4** *The main effect analysis of* *different levels of sanguinarin on the relative weight of ovary (g/kg live body weight) and ovarian differential follicle numbers in laying hens fed different levels of crude protein (CP)*

|  |  | | | | | | | | | | Probability | | |
| --- | --- | --- | --- | --- | --- | --- | --- | --- | --- | --- | --- | --- | --- |
|  | CP (%) | | |  | Sanguinarine (mg/kg) | | |  |  | CP | | Sanguinarine | SEM^b^ |
|  | 100 | 92.5 | 85 |  | 0.00 | 3.75 | 7.50 |  |  |  | |  |  |
| Ovary relative weight | 0.55 | 0.51 | 0.52 |  | 0.49 | 0.51 | 0.59 |  |  | 0.707 | | 0.381 | 0.063 |
| LWF^a^ (2–5 mm) | 35.6 | 32.8 | 31.2 |  | 36.3^a^ | 33.7^ab^ | 29.6^b^ |  |  | 0.211 | | 0.049 | 2.090 |
| SYF^a^ (5–10 mm) | 18.6 | 16.7 | 15.9 |  | 18.7 | 16.8 | 15.6 |  |  | 0.474 | | 0.398 | 2.281 |
| LYF^a^ (>10 mm) | 5.75 | 5.5 | 5.25 |  | 5.92 | 5.42 | 5.17 |  |  | 0.531 | | 0.309 | 0.520 |

^a^LYF: large yellow follicles; SYF: small yellow follicles; LWF: large white follicles

^b^SEM: standard error of the mean (n= 8 birds).

^a-b^ Means with no common superscript within each row are significantly (P<0.05) different.

**Table S5** *The main effect analysis of* *different levels of sanguinarin on illeal nutrient digestibility in laying hens fed different levels of crude protein (CP)*

|  |  | | | | | | | | | | Probability | | |
| --- | --- | --- | --- | --- | --- | --- | --- | --- | --- | --- | --- | --- | --- |
|  | CP (%) | | |  | Sanguinarine (mg/kg) | | |  |  | CP | | Sanguinarine | SEM^a^ |
|  | 100 | 92.5 | 85 |  | 0.00 | 3.75 | 7.50 |  |  |  | |  |  |
| DM^b^ digestibility (%) | 65.8^a^ | 64.3^ab^ | 62.4^b^ |  | 62.8^b^ | 64.7^ab^ | 65.0^a^ |  |  | 0.011 | | 0.031 | 0.724 |
| CP digestibility (%) | 67.9 | 66.3 | 64.8 |  | 64.5^b^ | 66.7^ab^ | 67.8^a^ |  |  | 0.071 | | 0.047 | 0.971 |

^a^SEM: standard error of the mean (n= 8 birds).

^b^DM: dry matter.

^a-b^ Means with no common superscript within each row are significantly (P<0.05) different.

**Table S6** *The main effect analysis of* *different levels of sanguinarin on performance in laying hens fed different levels of crude protein (CP)*

|  |  | | | | | | | | | | Probability | | |
| --- | --- | --- | --- | --- | --- | --- | --- | --- | --- | --- | --- | --- | --- |
|  | CP (%) | | |  | Sanguinarine (mg/kg) | | |  |  | CP | | Sanguinarine | SEM^a^ |
|  | 100 | 92.5 | 85 |  | 0.00 | 3.75 | 7.50 |  |  |  | |  |  |
| Egg production (%) |  |  |  |  |  |  |  |  |  |  | |  |  |
| 1-35 d | 85.5^a^ | 83.4^ab^ | 80.7^b^ |  | 82.0^b^ | 83.1^ab^ | 84.5^a^ |  |  | 0.039 | | 0.046 | 1.132 |
| 36-70 d | 83.3 | 80.6 | 79.8 |  | 80.0 | 81.6 | 82.1 |  |  | 0.104 | | 0.482 | 1.775 |
| 1-70 d | 84.4 | 82.0 | 80.3 |  | 81.0 | 82.3 | 83.3 |  |  | 0.062 | | 0.097 | 1.313 |
| Egg weight (g) |  |  |  |  |  |  |  |  |  |  | |  |  |
| 1-35 d | 63.3 | 62.3 | 61.7 |  | 61.4 | 62.6 | 63.4 |  |  | 0.392 | | 0.321 | 1.315 |
| 36-70 d | 65.0 | 63.9 | 63.2 |  | 64.1 | 63.9 | 64.7 |  |  | 0.613 | | 0.864 | 1.903 |
| 1-70 d | 64.1 | 63.1 | 62.5 |  | 62.7 | 63.2 | 64.0 |  |  | 0.572 | | 0.651 | 1.758 |
| Egg mass (g/d per bird) |  |  |  |  |  |  |  |  |  |  | |  |  |
| 1-35 d | 54.1 | 51.9 | 49.8 |  | 50.3 | 52.0 | 53.6 |  |  | 0.059 | | 0.103 | 1.531 |
| 36-70 d | 54.1 | 51.5 | 50.5 |  | 51.3 | 52.2 | 53.1 |  |  | 0.092 | | 0.425 | 1.551 |
| 1-70 d | 54.1 | 51.8 | 50.1 |  | 50.8 | 52.1 | 53.3 |  |  | 0.109 | | 0.394 | 1.835 |
| Feed intake (g/d per bird) |  |  |  |  |  |  |  |  |  |  | |  |  |
| 1-35 d | 96.7 | 94.9 | 94.3 |  | 94.3 | 95.6 | 96.0 |  |  | 0.169 | | 0.471 | 1.153 |
| 36-70 d | 93.4 | 92. 6 | 92.0 |  | 92.6 | 92.1 | 93.2 |  |  | 0.691 | | 0.625 | 1.910 |
| 1-70 d | 95.1 | 93.7 | 93.2 |  | 93.5 | 93.9 | 94.6 |  |  | 0.375 | | 0.599 | 1.625 |
| FCR^b^ (g of feed: g of egg) |  |  |  |  |  |  |  |  |  |  | |  |  |
| 1-35 d | 1.79 | 1.83 | 1.89 |  | 1.88 | 1.84 | 1.79 |  |  | 0.194 | | 0.218 | 0.062 |
| 36-70 d | 1.73 | 1.8 | 1.82 |  | 1.81 | 1.77 | 1.76 |  |  | 0.406 | | 0.728 | 0.081 |
| 1-70 d | 1.76 | 1.82 | 1.86 |  | 1.85 | 1.81 | 1.78 |  |  | 0.325 | | 0.409 | 0.050 |

^a^SEM: standard error of the mean (n= 20 birds).

^b^FCR: feed conversion ratio.

^a-b^ Means with no common superscript within each row are significantly (P<0.05) different.
